# A Loss of Function in LprG−Rv1410c Homologues Attenuates Growth during Biofilm Formation in *Mycobacterium smegmatis*

**DOI:** 10.3390/pathogens12121375

**Published:** 2023-11-21

**Authors:** Lisa-Marie Nisbett, Mary L. Previti, Jessica C. Seeliger

**Affiliations:** Department of Pharmacological Sciences, Stony Brook University, 100 Nicolls Road, Stony Brook, NY 11794, USA

**Keywords:** mycobacteria, cell wall, biofilms, lipid transport, MmpL11, LprG, Rv1410c, gene expression

## Abstract

MmpL (mycobacterial membrane protein large) proteins are integral membrane proteins that have been implicated in the biosynthesis and/or transport of mycobacterial cell wall lipids. Given the cellular location of these proteins, however, it is unclear how cell wall lipids are transported beyond the inner membrane. Moreover, given that mycobacteria grow at the poles, we also do not understand how new cell wall is added in a highly localized and presumably coordinated manner. Here, we examine the relationship between two lipid transport pathways associated with the proteins MmpL11 and LprG−Rv1410c. The lipoprotein LprG has been shown to interact with proteins involved in cell wall processes including MmpL11, which is required in biofilms for the surface localization of certain lipids. Here we report that deletion of *mmpL11* (*MSMEG_0241*) or the *lprG−rv1410c* operon homologues *MSMEG_3070−3069* in *Mycobacterium smegmatis* produced similar biofilm defects that were distinct from that of the previously reported *mmpL11* transposon insertion mutant. Analysis of pellicle biofilms, bacterial growth, lipid profiles, and gene expression revealed that the biofilm phenotypes could not be directly explained by changes in the synthesis or localization of biofilm-related lipids or the expression of biofilm-related genes. Instead, the shared biofilm phenotype between Δ*MSMEG_3070−3069* and Δ*mmpL11* may be related to their modest growth defect, while the origins of the distinct *mmpL11::Tn* biofilm defect remain unclear. Our findings suggest that the mechanisms that drive pellicle biofilm formation in *M. smegmatis* are not connected to crosstalk between the LprG−Rv1410c and MmpL11 pathways and that any functional interaction between these proteins does not relate directly to their lipid transport function.

## 1. Introduction

Tuberculosis remains one of the top infectious killers worldwide [1]. Additionally, the continued emergence and spread of multidrug-resistant TB further demonstrates the urgent need for novel therapeutic approaches against *Mycobacterium tuberculosis* (*Mtb*), the causative agent of tuberculosis (TB) in humans. The drug resistance of *Mtb* is due in part to a well-armored cell wall, which is unique in composition and architecture. The outer membrane consists of an inner leaflet of very long chain mycolic acids covalently bound to an underlying arabinogalactan-peptidoglycan layer, and an outer leaflet composed of diverse noncovalently associated lipids such as trehalose 6,6′-dimycolate (TDM), phthiocerol dimycocerosate (PDIM), and sulfolipids [2,3]. Because its structure is distinct from the outer membrane of Gram-negative bacteria, this outer layer is commonly referred to as the mycomembrane.

Mycomembrane lipids are positioned to play important roles in intercellular interactions. Indeed, disruptions in lipid biosynthesis or trafficking attenuate pathogenesis, as has been reviewed elsewhere [4,5,6]. Perturbations in mycobacterial lipids are also correlated with defects in the formation of biofilms [7,8,9,10,11,12,13]. Broadly defined, biofilms are communities of microorganisms that are associated with a self-secreted extracellular matrix that is thought to serve structural and communication roles [14]. These communities can range from surface-attached colonies to pellicles at the air–liquid interface, with the latter being one of the most studied models for mycobacterial biofilms [15]. Importantly, several mycobacterial species have been shown to form biofilms in the clinical setting. The fast-growing non-tuberculous mycobacterium (NTM) *M. abscessus* forms biofilms within the thickened alveolar walls and airways of cystic fibrosis patients [16], as well as in the lung cavity of a patient with chronic obstructive pulmonary disease [17]. Yamazaki et al. demonstrated a role for the biofilms of another NTM, *M. avium*, in bronchiolar and bronchial infections [18]. *Mtb* was recently found to form biofilms in human lung samples with TB, and in this state *Mtb* was protected from chemotherapeutic agents [19]. Moreover, mycobacterial species in this biofilm state have been found to be more resistant to antibiotics [20,21]. Overall, the relevance of biofilms to clinical infections and treatment motivates investigating the genetic and molecular mechanisms by which they form.

Diverse factors drive mycobacterial biofilm formation, as has been reviewed elsewhere [22,23,24] and in detail for multiple mycobacterial species by Chakraborty and Kumar [25]. For the two most highly studied species, *M. tuberculosis* and *M. smegmatis,* the most consistent association has been with lipids, including free mycolic acids (FMA) [11,13], mycolyl diacylglycerides (MDAG) [26], monomeromycolyl diacylglycerides (MMDAG) [7,27], mycolate wax esters (MWE) [7,8,27] and, in *M. smegmatis*, glycopeptidolipids (GPL) [9,28]. Genetic alterations associated with changes in lipids can affect stages that are commonly defined for bacterial biofilms and are shared by mycobacteria: substratum attachment, intercellular aggregation, and architecture maturation. For example, in *M. smegmatis*, *lsr2* disruption is required for intracellular aggregation, whereas *groEl1* is required for maturation. Further, *groEL1* has an unexpected role in stabilizing FAS II enzymes KasA/KasB, whose activity feeds into mycolic acid biosynthesis; the loss of *groEL1* leads to the production of short chain mycolates (thought to be precursors to FMAs) [12]. The loss of *lsr2* is suppressed by mutations in the GPL biosynthesis gene *mps* [29]. This contradicts studies showing that GPL is required for biofilm formation [9,28], but suggests that this process depends on the integration and timing of diverse factors, and that any one factor, such as a single lipid class, cannot necessarily serve as a universal marker. Indeed, other studies have identified correlations with assorted genes and lipids: Loss of function in enzymes that make the second messengers (pp)pGpp (*rel*) or c-di-GMP (*dcpA*) are defective for biofilm formation and show reductions in total levels of not only GPLs, but also other polar lipids [30]. Notably, the involvement of *lsr2* in biofilms was uncovered via a transposon insertion library screen in *M. smegmatis* [26]. Additional prospective studies have sought to identify other factors that influence mycobacterial biofilm formation through not only transposon insertion library screens [10,31,32] in other species, but also gene expression profiling [10,33,34], and comparative proteomics [35,36]. These have further underscored the association of lipids with the identification of additional genes and proteins related to lipid biosynthesis.

Overall, the correlations between membrane lipid composition and biofilm formation highlight lipid biosynthesis and transport as targets for disrupting these antibiotic-resistant cell aggregates. Many of the enzymes that produce mycobacterial lipids have been identified, but the mechanisms by which these products reach the cell wall and mycomembrane remain poorly understood. Of the few pathways that have been characterized, the lipoprotein LprG and the co-cistronic integral membrane transporter Rv1410c are conserved across mycobacteria and are associated with the transport of triacyl lipids such as triacylglyceride and lipoarabinomannan [37,38,39]. Intriguingly, LprG has been shown to interact in vitro with the periplasmic domains of MmpL11 [40], which is involved in biofilm maturation and the surface localization of mycolic acid-containing lipids in biofilms such as MWE and MMDAG [7,27]. This evidence for a physical interaction led us to investigate the possible functional interaction between these pathways.

In this study, our aim was to investigate the potential overlapping roles of LprG−Rv1410c and MmpL11 in lipid transport and biofilm formation. Based on previous work, we focused these studies on *M. smegmatis* and the effects of disrupting the homologues *MSMEG_3070−3069* (*lprG−rv1410c*) and *mmpL11* (*MSMEG_0241*; based on prevailing nomenclature, we use *mmpL11* to designate both the *M. smegmatis* and *Mtb* homologues throughout). We found that the targeted deletion of *mmpL11* or *MSMEG_3070−3069* led to similar biofilm defects that were distinct from that of the previously reported *mmpL11* transposon insertion mutant [7] and were not correlated with shared changes in the total or cell-surface levels of biofilm-related lipids. Instead, we found that differences in *mmpL11* gene expression could underlie the distinct biofilm phenotype between Δ*mmpL11* and *mmpL11::Tn*, and a modest growth defect could explain the biofilm defect shared between Δ*MSMEG_3070−3069* and Δ*mmpL11*. Our findings thus suggest that although LprG and MmpL11 may physically interact in vitro [40], the biofilm defects in the Δ*MSMEG_3070−3069*, Δ*mmpL11*, and *mmpL11::Tn* strains are not due to overlapping functions of these loci in lipid transport.

## 2. Materials and Methods

### 2.1. Bacterial Strains, Culture Media, and Culture Conditions

Bacterial strains used in this study are listed in Table S1. *Mycobacterium smegmatis* mc^2^155 served as the parent (wild-type) strain. For planktonic growth, *M. smegmatis* was cultured at 37 °C with agitation at 250 rpm in Middlebrook 7H9 broth supplemented with 10% albumin/dextrose/catalase (ADC), 0.5% glycerol, and 0.05% Tween 80 (all % are *v/v* unless otherwise indicated). For growth on solid medium, unless otherwise indicated, *M. smegmatis* was plated on Middlebrook 7H11 agar containing 10% ADC, 0.5% glycerol, and 0.05% Tween 80. When required, hygromycin, kanamycin, and/or zeocin were added to the growth medium at 50, 25, or 10 µg/mL, respectively.

### 2.2. Construction of Mutant and Complement M. smegmatis Strains

All primers and plasmids used in the construction of mutant and complement strains of *mmpL11* are listed in Table S2. The Δ*MSMEG_3070−3069*, Δ*MSMEG_3070−3069::lprG*, Δ*MSMEG_3070−3069::rv1410c*, Δ*MSMEG_3070−3069::lprG−rv1410c* and wild-type parent *M. smegmatis* strains were a gift from Eric Rubin [39,41]. The *mmpL11::Tn*, *mmpL11::Tn::mmpL11_Msm_*, *mmpL11::Tn::mmpL11_Mtb_* and wild-type parent *M. smegmatis* strains were a gift from Georgiana Purdy [7]. A Δ*mmpL11* (Δ*MSMEG_0241*) strain was generated via recombineering [42] (Figure S1A). Briefly, 125 bp fragments upstream and downstream of *mmpL11* were PCR-amplified from genomic *M. smegmatis* mc^2^155 DNA using primers omlp741, omlp742, omlp743, and omlp744, and the products were inserted into the pJSC407 plasmid (a gift from Jeffrey Cox) flanking a hygromycin resistance gene. The resulting plasmid pMLP082 was sequence-verified. The recombineering substrate was prepared via PCR amplification using primers omlp741 and omlp744 and gel purified. Wild-type mc^2^155 containing the pNIT-RecET-SacB-Kan plasmid (a gift from Christopher Sassetti) was cultured to OD_600_ ~0.7 and the recombinase was then induced for 3 h with 10 µM isovaleronitrile. Electrocompetent cells prepared via several washes with 10% (*v*/*v*) sterile glycerol were electroporated with 1 µg of recombineering substrate and plated on 7H11/10% ADC/hygromycin agar plates to select for recombinants. Colonies were screened for recombination using PCR primers omlp745, ojcs240, omlp741, and omlp744. A confirmed clone was cured of the pNIT-RecET-SacB-Kan plasmid by screening for hygromycin resistance and kanamycin sensitivity on 7H11/10% ADC agar plates containing the appropriate antibiotic.

For complementation, the *M. smegmatis mmpL11* gene was amplified using primers omlp745 and omlp746, and cloned into pMV306 using In-Fusion cloning (Takara Bio, Mountain View, CA, USA). The resulting plasmid pMLP083 was sequence-verified and subsequently electroporated into Δ*mmpL11* to generate Δ*mmpL11::mmpL11_Msm_*.

### 2.3. Biofilm Growth and CFU Enumeration

For biofilm assays, *M. smegmatis* strains were cultured as previously described [7]. Briefly, *M. smegmatis* was inoculated at OD_600_ 0.05 in Sauton’s medium, without Tween 80, in polystyrene Petri dishes (100 mm × 15 mm) and incubated at 30 °C without disturbance for up to 5 days. Sauton’s medium contained 0.5 g/L monobasic potassium phosphate, 0.5 g/L anhydrous magnesium sulfate, 4.0 g/L L-asparagine, 0.05 g/L ferric ammonium citrate, 2.0 g/L anhydrous citric acid, 4.76% glycerol, and 1 mg/L zinc sulfate heptahydrate at pH 7.0. To enumerate viable bacteria grown under biofilm-inducing conditions, pellicle biofilms were harvested 2 days or 4 days after inoculation via centrifugation at 3000× *g* for 10 min. Pellets were resuspended in 5 mL PBS with 0.1% Tween 80, and 1 mL of 3 mm glass beads (Fisher, Boston, MA, USA) were added to mechanically disrupt the biofilms via manual agitation. The bacteria were then further dispersed by passing the cells through a tuberculin syringe (BD; REF 309626) seven times. Following serial dilution in PBS/0.1% Tween 80, 100 µL of each dilution (10^−6^ to 10^−9^) was plated on Middlebrook 7H10/10% ADC/0.05% glycerol agar plates. Colony forming units (CFU) were enumerated after 3 days and statistical analyses were performed using GraphPad Prism version 10.

### 2.4. Lipid Extraction and Analysis

“Harsh” surface lipid extraction was performed as previously described [27]. Briefly, pellicle biofilms were harvested 5 days after inoculation via centrifugation at 3000× *g* for 10 min, resuspended in 5 mL hexanes and sonicated at 55 °C for 15 min. The extracts were then clarified via centrifugation at 1000× *g* for 10 min and dried under nitrogen gas at 30 °C (Biotage TurboVap LV). For “gentle” surface lipid extraction, pellicle biofilms were harvested 5 days after inoculation via centrifugation at 3000× *g* for 10 min, resuspended in 5 mL hexanes and vortexed for 30 s. The extracts were then clarified via centrifugation at 1000× *g* for 10 min and the supernatants were dried under nitrogen gas at 30 °C (Biotage TurboVap LV). To extract total lipids, the same procedure as gentle hexane extraction was used, except that the harvested biofilms were resuspended in 5 mL of chloroform/methanol (2:1, *v*/*v*).

For analysis via thin-layer chromatography, surface and total lipid extracts were resuspended in chloroform/methanol (2:1, *v*/*v*) and spotted onto silica plates (MilliporeSigma, Chicago, IL, USA 1.05553.0001). Loads were normalized according to dry weight. Free mycolates (FM) were resolved with chloroform/methanol (96:4, *v*/*v*) [11]. Mycolate wax ester (MWE) and monomeromycolyl diacylglycerol (MMDAG) were resolved with toluene/acetone (99:1, *v*/*v*) [7]. Trehalose dimycolate (TDM), trehalose monomycolate (TMM), phosphatidylethanolamine (PE), phosphatidylinositol (PI), cardiolipin (CL), and phosphatidylinositolmannosides (PIMs) were resolved with chloroform/methanol/water (30:8:1 *v*/*v*) [43]. Lipids were visualized via immersion in 10% phosphomolybdic acid in ethanol and charred via heating. The amounts of FM, MWE, MMDAG, TDM, TMM, PE, PI, CL, and PIMs, as a percentage of the total signal for each sample, were calculated via densitometry using ImageJ [44], and the mean ± standard deviation (S.D.) from three independent experiments were analyzed. Statistical analysis was performed using GraphPad Prism version 10.

### 2.5. RNA Extraction and RT-qPCR

All oligonucleotides used for RT-qPCR are listed in Table S2. Pellicle biofilms were harvested 5 days after inoculation via centrifugation at 3000× *g* for 10 min and the pellets were resuspended in 1 mL TRIzol LS (Thermo Fisher Scientific, Waltham, MA, USA) and stored at −80 °C. TRIzol resuspensions were thawed on ice and subsequently lysed via bead beating (BeadRupter 12, OMNI International, Kennesaw, GA, USA) using 0.1 mm zirconia/silica beads (Biospec, Bartlesville, OK, USA) for 30 s at 6 m/s followed by 5 min incubation on ice for a total of 4 cycles. The lysates were clarified via centrifugation at 12,000× *g* at 4 °C for 10 min and total RNA was extracted and purified from the aqueous phase using chloroform/isoamyl alcohol (24:1, *v*/*v*) and the Qiagen Rneasy kit, respectively. Successful isolation of total RNA was confirmed via visual inspection using 2% agarose gel electrophoresis. DNA contamination was removed using the TurboDNAse free kit (Thermo Fisher Scientific, Waltham, MA, USA) and 1 µg of RNA was used for cDNA synthesis with the Verso cDNA synthesis kit (Thermo Fisher Scientific, Waltham, MA, USA) following the manufacturer’s instructions. RT-qPCR was performed on a LightCycler 480 (Roche, Indianapolis, IN, USA) with SYBR Green Master mix (Thermo Fisher Scientific, Waltham, MA, USA) following the manufacturer’s instructions. Relative expression of target genes was calculated using the Pfaffl method [45].

## 3. Results and Discussion

### 3.1. Loss of MSMEG_3070 3069 or mmpL11 Leads to Similar Defects in Biofilm Formation 

**Figure 1:**
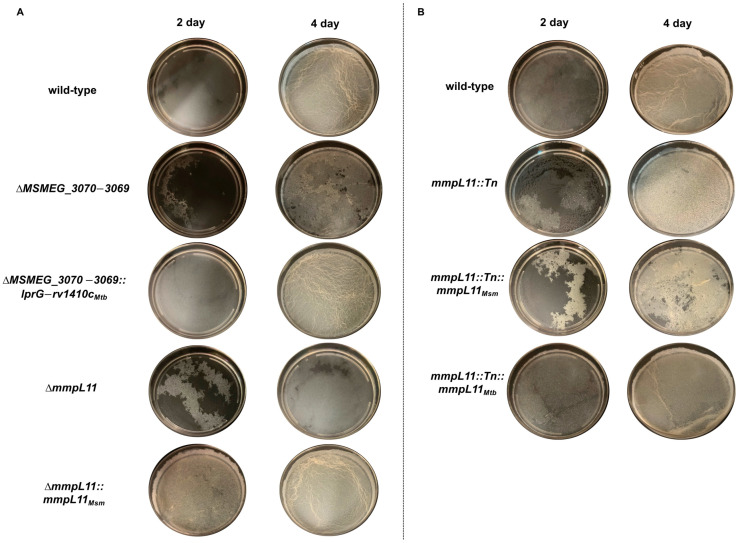
*MSMEG_3070−3069* and *mmpL11* mutants displayed impaired biofilm formation. (**A**,**B**) Pellicle biofilm formation at the air–liquid interface at 2 and 4 days after inoculation for (**A**) parent wild-type, Δ*MSMEG_3070−3069*, Δ*mmpL11*, and associated complement strains and (**B**) parent wild-type, mmpL::Tn, and associated complement strains. Equal numbers of bacteria were inoculated in Sauton’s medium, without Tween 80, in polystyrene dishes and the plates were incubated at 30 °C without disturbance for 5 days. Data shown are representative of three biological replicates.

We hypothesized that if LprG−Rv1410c functions parallel to or downstream of MmpL11, loss of function would lead to a similar defect in biofilm formation. To test this, we used a previously reported *M. smegmatis* Δ*MSMEG_3070−3069* deletion strain [39,41]. To enable direct comparison, we constructed a targeted deletion of *mmpL11* in the same parent wild-type strain. Following established protocols [7], we compared the development of pellicle biofilms over a total of 5 days for these and corresponding complement strains (Figure 1A and Figure S2). The wild-type strain formed an immature pellicle biofilm at the air–liquid interface after 2 days; a monolayer-like pellicle biofilm after 3 days; and a mature biofilm with reticulations after 4 days. In contrast, both Δ*MSMEG_3070−3069* and Δ*mmpL11* showed a delay in pellicle biofilm formation and failed to form mature reticulated biofilms by day 4 (Figure 1A) or even day 5 (Figure S2). Importantly, both the kinetics and full maturation of biofilm formation were restored via complementation (Figure 1A and Figure S2). Since an in vitro interaction was previously detected between MmpL11 and LprG, but not with Rv1410c [40], we also assayed biofilm formation in Δ*MSMEG_3070−3069,* complemented with either *Mtb lprG* or *rv1410c,* rather than the full operon (Figure S3). Both singly complemented strains showed an identical phenotype to the wild-type strain, suggesting that either gene is sufficient for wild-type biofilm formation but only removal of both genes leads to a biofilm phenotype that is similar to that of Δ*mmpL11*.

**Figure 2:**
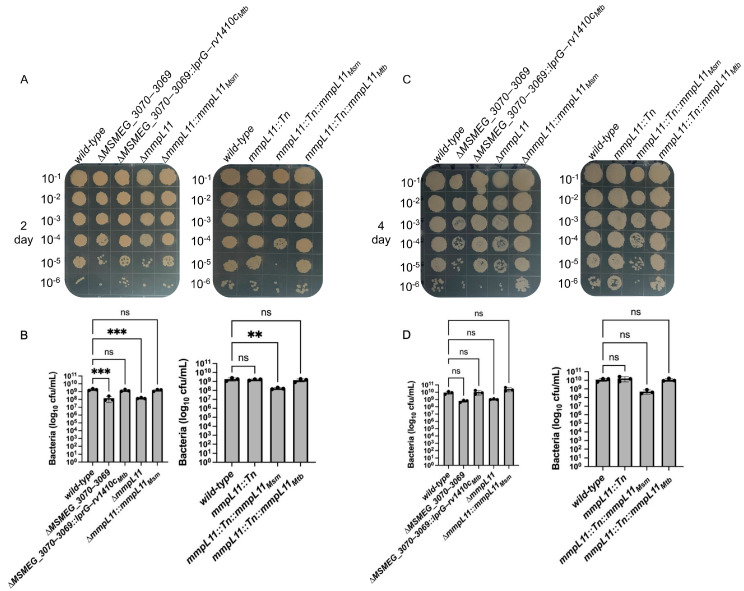
*MSMEG_3070−3069* and Δ*mmpL11* show delayed growth compared to the wild-type during biofilm formation. Growth was assessed (**A**,**B**) 2 days and (**C**,**D**) 4 days after biofilm inoculation by (**A**,**C**) spotting serial dilutions on 7H10/ADC/glycerol agar or (**B**,**D**) enumerating CFU. For (**A**,**C**), data representative of three independent experiments are shown. For (**B**,**D**), the data shown are the mean ± S.D. of three independent experiments. Statistical significance was determined using one-way ANOVA (Dunnett’s test). (**, *p* = 0.0021; ***, *p* = 0.0002; ns: not significant).

The delayed maturation biofilm phenotype prompted us to ask whether the gene deletion strains were attenuated for growth under biofilm conditions. Indeed, we found that both Δ*MSMEG_3070*−*3069* and Δ*mmpL11* strains grew more slowly, with approximately 1-log fewer colony forming units (CFU) compared to the wild-type strain 2 days after inoculation (Figure 2A,B). Although this difference was not statistically significant 4 days after inoculation, the trend and magnitude were similar (Figure 2C,D). For both mutants, growth at both timepoints was restored via complementation (Figure 2A,C). Thus, both Δ*MSMEG_3070−3069* and Δ*mmpL11* display a moderate growth defect that may contribute to the observed biofilm defect.

This contrasts with *M. smegmatis mmpL11::Tn*, which was reported to have a biofilm maturation defect, but wild-type-like growth under similar conditions [7]. Therefore, we also assayed the biofilm and growth characteristics of the transposon insertion and corresponding complement strains. For *mmpL11::Tn*, biofilm formation resembled that of the parent wild-type strain until day 3 (Figure 1B and Figure S2), after which it remained an easily disturbed monolayer without reticulations (Figure 1B and Figure S2), and its growth was similar to that of the wild-type at both 2 days and 4 days post inoculation (Figure 2). While this characterization is similar to previous reports [7], it is notably distinct from the phenotype of our Δ*mmpL11*-targeted deletion strain, suggesting that the different methods of gene disruption have different consequences for biofilm formation and growth. Notably, *mmpL11* is expressed in an operon upstream of *MSMEG_0240*, and transposon insertion could have different effects on *MSMEG_0240* expression than gene deletion, although how expression changes would affect bacterial physiology is not obvious from the available information: *MSMEG_0240* is annotated as a transcription factor, but is otherwise an uncharacterized hypothetical protein.

Finally, in our hands and in contrast to previous reports [7], complementation of *mmpL11::Tn* with *mmpL11* from *M. smegmatis* did not restore biofilm formation and growth trended lower compared to the wild-type (Figure 1, Figure 2 and Figure S2). In contrast, the *M. tuberculosis mmpL11* complement yielded wild-type-like biofilms and growth. Differences in how strains were complemented complicate the interpretation of these conflicting results. Complementation of *mmpL11::Tn* was achieved using a multi-copy plasmid with a strong constitutive promoter driving expression of *M. smegmatis mmpL11*, but in the *Mtb mmpL11* complement, *mmpL11* gene expression was driven by a native promoter from a single-copy integrating plasmid. Further, we complemented Δ*mmpL11* from a single-copy integrated plasmid with a native promoter driving expression of *Msm mmpL11*. Overall, the parallels in biofilm formation and growth between Δ*MSMEG_3070−3069* and Δ*mmpL11*, but contrast with *mmpL11::Tn*, led us to examine other aspects of these strains that could underlie these similarities and differences.

### 3.2. MSMEG_3070−3069 Is Not Required for the Surface Localization of Mycolate Wax Esters and Monomeromycolyl Diacylglycerides

The defect in biofilm formation in the *mmpL11::Tn* strain has been correlated with a biofilm- and cell surface-specific loss of certain lipids: mycolate wax esters (MWE), monomeromycolyl diacylglyceride (MMDAG), and a chromatographically resolved but chemically undefined species annotated as Lipid A [7]. This phenotype led to the assignment of MmpL11 as the transporter for these lipid classes. Changes in other mycomembrane lipids, such as disruptions in TDM synthesis and enzymatic hydrolysis by trehalose dimycolate hydrolase [11], have also been associated with biofilm defects. These results suggest a model in which protein interactions involving lipid biosynthesis enzymes modulate lipid composition and thereby, biofilm formation. Based on this model, the physical interaction between LprG and MmpL11 could modulate the transport of MmpL11-associated lipids like MWE and MMDAG and thereby affect biofilm formation. We would thus predict that Δ*MSMEG_3070−3069* and Δ*mmpL11* have similar changes in their surface lipids compared to the wild-type, although, based on biofilm formation and growth, possibly distinct from *mmpL11::Tn*. To test this hypothesis, we analyzed lipids extracted from *M. smegmatis* biofilms 5 days post inoculation via thin layer chromatography, thus allowing for the comparison of lipid profiles with the biofilm phenotypes shown in Figure 1A and Figure S2.

**Figure 3:**
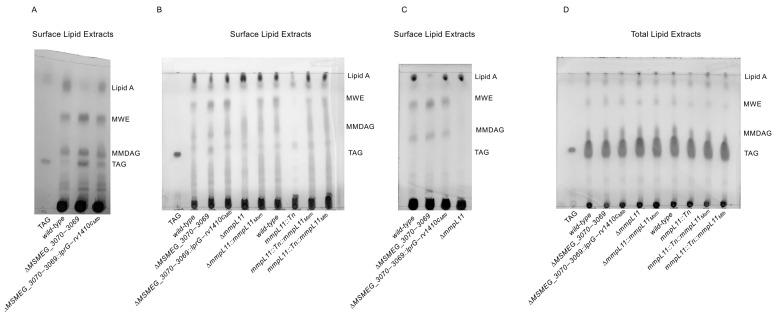
*MSMEG_3070−3069* mutant has an altered “harsh” hexane extract surface- exposed lipid profile of Lipid A, MWE, MMDAG and TAG but does not have an altered “gentle” hexane extract surface-exposed lipid profile. (**A**) 195 µg of surface lipid extracts, (**B**,**C**) 200 µg of surface lipid extracts and (**D**) 200 µg of total lipid extracts were resolved by TLC in toluene:acetone (99:1, *v*/*v*). Tripalmitate (labeled as TAG) was used as a migration standard. Lipid A, MWE and MMDAG were assigned by comparison to previously published TLCs performed under the same conditions [7,27]. TLC plates were immersed in 10% phosphomolybdic acid and charred to visualize lipids. The line near the top of the plates indicates the solvent front. Data shown represents one biological replicate.

Surface-selective loss of MWE, MMDAG, and lipid A in *mmpL11::Tn* biofilms was previously shown by extracting lipids with hexanes and sonication with heating. To facilitate direct comparison, we applied this method to Δ*MSMEG_3070−3069* and the corresponding wild-type and complement strains. Surprisingly, Δ*MSMEG_3070−3069* extracts showed elevated triacylglycerides (Figure 3A). This result contradicts the prevailing model from *Mtb,* in which LprG/Rv1410c transport TAG to the mycomembrane, but is consistent with the accumulation of select TAG isoforms in the total lipid extracts of *Mtb* Δ*lprG−rv1410c*. One possible explanation is that the extraction method is not surface-selective: sonication was performed at 50 °C [27], which, may have led to the solubilization of lipids deeper within the mycobacterial cell envelope. We thus compared this to a gentler extraction in hexanes without sonication or heating, an approach that has been previously used to characterize lipid transport in *Mtb* [46,47,48]. Although TAG is considered a mycomembrane lipid based on surface-selective lipid extraction with reverse micelles, hexanes extracts obtained without sonication or heating showed markedly reduced extraction of TAGs, based on the comparison between treatment conditions and using a commercial tripalmitate standard (Figure 3B). However, when applied to *mmpL11::Tn*, this gentler extraction of hexanes still consistently recapitulated the previously reported loss of MWE, MMDAG, and lipid A (Figure 3B). We concluded that while this method may not exhaustively extract all surface lipids, it was more selective and therefore preferred for further analyses since it was less likely to lead to a false negative lipid transport phenotype.

We therefore proceeded to analyze selective “gentle” hexanes and total chloroform-methanol extracts for all *MSMEG_3070−3069*- and *mmpL11*-associated strains (Figure 3). For all strains, no major differences were detected in the total cellular levels of MWE, MMDAG, or lipid A (Figure 3D). In contrast, hexanes extracts revealed distinctions between the strains that did not, however, correlate with biofilm phenotypes (Figure 1, Figure 3B,C and Figure S2). First, *M. smegmatis mmpL11* complementation restored MWE, MMDAG, and lipid A levels in *mmpL11::Tn*. Second, Δ*mmpL11* only partially recapitulated *mmpL11::Tn*: While a reduction in MWE was evident, changes in MMDAG or lipid A were not, and this phenotype was restored via complementation with *Msm mmpL11*. (Because MMDAG is not well resolved in the replicate shown in Figure 3B, a replicate with a subset of strains more clearly illustrating the reduction of MMDAG for Δ*mmpL11* is provided in Figure 3C.) Finally, the loss of *MSMEG_3070 3069* function did not strongly affect any of the three lipid classes. These results suggest that changes in the surface localization of MWE, MMDAG, and lipid A can broadly be correlated with the loss of *mmpL11* function, but not the loss of *MSMEG_3070−3069* function. Consequently, the shared defect in biofilm formation cannot be explained by the changes in the surface localization of these three classes of lipids in these strains.

**Figure 4:**
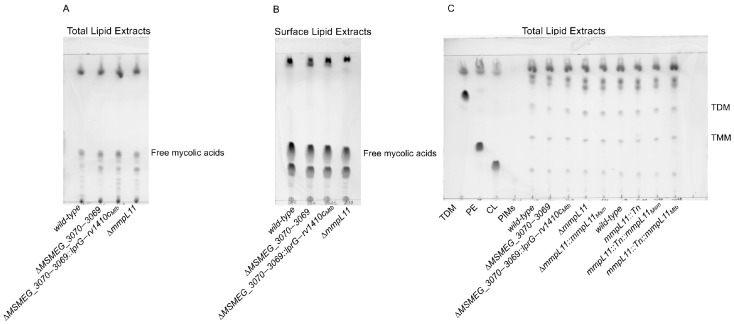
The free mycolic acid lipid profile in the total and surface lipid extracts, and the trehalose dimycolate (TDM) and trehalose monomycolate (TMM) lipid profiles in the total lipid extracts are not altered in any strain. (**A**) 50 µg total lipid and (**B**) 50 µg surface lipid extracts were resolved via TLC in a chloroform: methanol (96:4, *v*/*v*) solvent system. (**C**) 50 µg total lipid extracts were resolved via TLC in chloroform: methanol: water (30:8:1, *v*/*v*). TLC plates were immersed in 10% phosphomolybdic acid and charred to visualize lipids. The line near the top of the plate indicates the solvent front. TDM: trehalose dimycolate, TMM: trehalose monomycolate, PE: phosphatidylethanolamine, CL: cardiolipin, PIMs: phosphatidylinositolmannosides. Data shown represent one replicate.

As noted in the introduction, free mycolic acids (FMA) have been implicated in biofilm formation through changes in the mycolic acid synthesis (specifically, the modulation of KasA activity by interaction with the chaperone GroEL1 [12]) and incorporation into the extracellular matrix (through the activity of a trehalose dimycolate hydrolase [11]). To test whether changes in FMA underlie the shared biofilm phenotype, we also analyzed free mycolic acid and trehalose (di)mycolate levels in all *MSMEG_3070−3069* and *mmpL11*-related strains. However, no major changes in any of these lipid classes were detected (Figure 4). In summary we found no shared differences in surface nor total levels of lipids previously associated with biofilm formation that could explain the biofilm phenotypes we observed upon loss of *MSMEG_3070−3069* and *mmpL11*.

### 3.3. Loss of MSMEG_3070−3069 or mmpL11 Does Not Correlate with Changes in Expression of Genes Associated with Biofilm Formation

**Figure 5:**
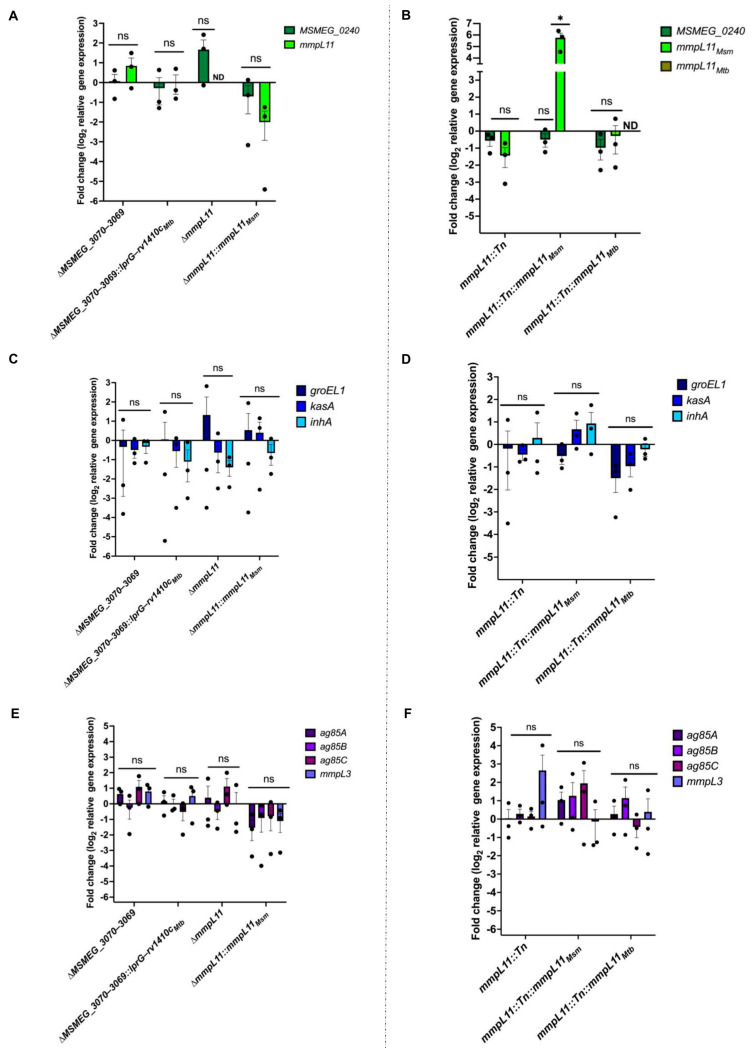
RT-qPCR analysis of genes connected to biofilm formation in M. smegmatis. RT-qPCR analysis of (**A**,**B**) *MSMEG_0240* and *mmpL11*, (**C**,**D**) *groEL1*, *kasA,* and *inhA* and (**E**,**F**) *ag85A*, *ag85B*, *ag85C*, and mmpL3 in the wild-type, mutant, and complement strains. Changes in gene expression are relative to the appropriate wild-type parent strain control and were calculated using the Pfaffl method [45]. The housekeeping gene *sigA* was used as the endogenous reference for normalization between samples. Data represent mean ± SEM of three independent biological replicates. Statistical significance was determined using one-way ANOVA (Dunnett’s test) in GraphPad Prism version 10. (*, *p* = 0.03; ns: not significant)**.** ND: not detected.

Since we found no shared differences in lipids previously associated with biofilm formation, we hypothesized that the biofilm defect in the Δ*MSMEG_3070−3069*, Δ*mmpL11,* and *mmpL11::Tn* strains could be due to changes in the expression of biofilm-related genes. To test this hypothesis, we measured the expression of *mmpL11*, the co-cistronic *MSMEG_0240,* and genes associated with biofilm maturation (*groEl1*, *kasA*) [12] or required for TDM synthesis (*mmpL3*, *ag85A*, *ag85B*, *ag85C*) [11]. Compared to the wild-type, no significant changes in gene expression were detected for any of these genes (Figure 5).

The only exception was *mmpL11*. As expected, expression was not detected in the targeted gene deletion Δ*mmpL11* (Figure 5A). Low levels were found in *mmpL11::Tn,* as assessed using a primer pair upstream of the mapped transposon insertion site, suggesting that the insertion suppressed but did not prevent transcription (Figure 5B). However, in *mmpL11::Tn* complemented with *M. smegmatis mmpL11*, the complement was highly expressed (~64-fold increase compared to the wild-type, an average of three independent experiments) (Figure 5B). This suggests that *M. smegmatis mmpL11* overexpression underlies the lack of biofilm complementation (Figure 1 and Figure S2) and the unexpected growth defect (Figure 2) in this strain. In contrast, *Mtb mmpL11* expression was not detected even though this strain showed complementation of the *mmpL11::Tn* biofilm defect (Figure 1, Figure 5B and Figure S2). It is possible that this message is unstable when isolated, or that even very low expression is sufficient for complementation. In summary, differences in *mmpL11* gene expression may contribute to the distinct biofilm phenotypes seen between Δ*mmpL11* and *mmpL11::Tn*, but cannot explain the biofilm defect shared between Δ*mmpL11* and Δ*MSMEG_3070−3069*.

## 4. Conclusions

Taken together, our findings suggest that the loss of function of *MSMEG_3070−3069* and *mmpL11* leads to a defect in biofilm formation that is distinct from that of a previously characterized *mmpL11* transposon insertion mutant in *M. smegmatis*. This work also revealed that neither the difference in the biofilm phenotype between Δ*mmpL11* and *mmpL11::Tn*, nor the similar biofilm defect in Δ*MSMEG_3070−3069* and Δ*mmpL11*, can be directly explained by changes in the total or cell-surface levels of lipid classes that have been correlated with biofilm formation, or by changes in the expression of genes associated with biofilm formation or the synthesis of biofilm-related lipids. Instead, both null strains show a moderate growth defect that may underlie the observed delay in biofilm formation. Given that the common growth defect is not correlated with shared changes in the lipid profile, it is possible that the disruption of lipid transport in these strains dysregulates lipid metabolism in ways that similarly compromise growth. This model has been proposed for *lprG−rv1410c* in *Mtb* [39], but remains to be experimentally explored. Overall, we found that while LprG and MmpL11 may physically interact [40], these proteins do not function in overlapping lipid transport pathways that correlate with biofilm formation.

Finally, the growth and the biofilm defects in Δ*mmpL11* were not shared by the *mmpL11::Tn* transposon mutant, but the two strains were otherwise similar in all other assays. The only clear difference was that the chemical undefined species lipid A was retained in hexanes extracts of Δ*mmpL11* (Figure 3B). While we showed that this change was not correlated with differences in expression of the downstream *MSMEG_0240*, mutations in unlinked genes elsewhere in the chromosome or strain-specific responses to genetic modification could be responsible. An alternative, but less likely, explanation is that the low-level transcription of *mmpL11* in *mmpL11::Tn* permits the expression of an N-terminal fragment that complements growth without restoring lipid phenotypes. Nevertheless, comparison of the two strains confirms that *mmpL11* is required in biofilms for the surface localization of MMDAG and MWE. Our data also provide a note of caution concerning complementation, as the multi-copy expression of *mmpL11* from a constitutive promoter led to significant overexpression and could underlie the unexpected phenotypes seen when compared to the parent strain.

## Data Availability

Data are contained within the article and supplementary materials.

## Supplementary Materials

The following supporting information can be downloaded at https://www.mdpi.com/article/10.3390/pathogens12121375/s1, Figure S1: schematic of plasmids used in the study and recombineering strategy used to generate Δ*mmpL11* strain; Figure S2: 5-day time course of pellicle biofilm formation at the air–liquid interface; Figure S3: 5-day time course of *MSMEG_3070−3069* mutant and complement strains’ pellicle biofilm formation at the air–liquid interface; Table S1: strains used in this study; Table S2: plasmids and primers used in this study.
