# Supplementary material for: A Loss of Function in LprG−Rv1410c Homologues Attenuates Growth during Biofilm Formation in Mycobacterium smegmatis
Source: Pathogens. 2023 Nov 21;12(12):1375. doi: 10.3390/pathogens12121375 (PMC10745849; doi:10.3390/pathogens12121375)
Supplement: Supplementary file 1 [file pathogens-12-01375-s001.zip › pathogens-2669097-supplementary.pdf]

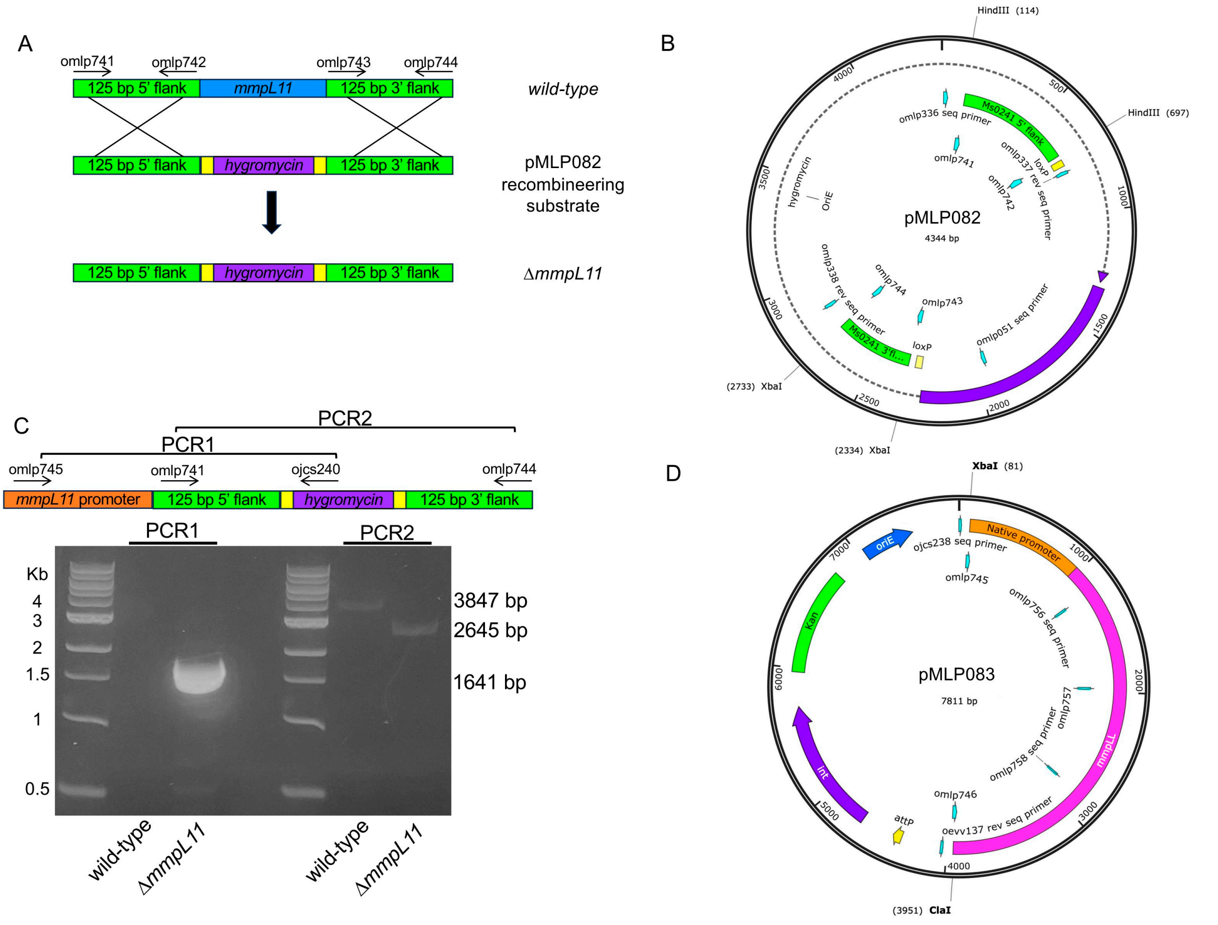

Figure S1. Generation and confirmation of *M. smegmatis* Δ*mmpL11*. (**A**) Schematic of the recombineering strategy for generating *M. smegmatis* Δ*mmpL11*. (**B**) Plasmid map for the pMLP082 plasmid used as the template for the recombineering substrate. (**C**) Schematic and results for PCR confirmation of recombination at the *mmpL11* locus. For PCR1 the predicted band size was 1641 bp. A corresponding product was observed only from Δ*mmpL11* genomic DNA, indicating that *mmpL11* was successfully replaced by the hygromycin antibiotic gene cassette. For PCR2 the expected band size was 3847 bp for the wild type and 2645 bp for Δ*mmpL11*. The expected shift in band PCR2 product size was observed for *ΔmmpL11* vs. the wild type. Δ*mmpL11* was further confirmed by sequencing the gel-purified PCR2 product from Δ*mmpL11*. (**D**) Plasmid map depicting the *mmpL11* complement construct (pMLP083). Plasmid maps in B and D were generated using SnapGene.

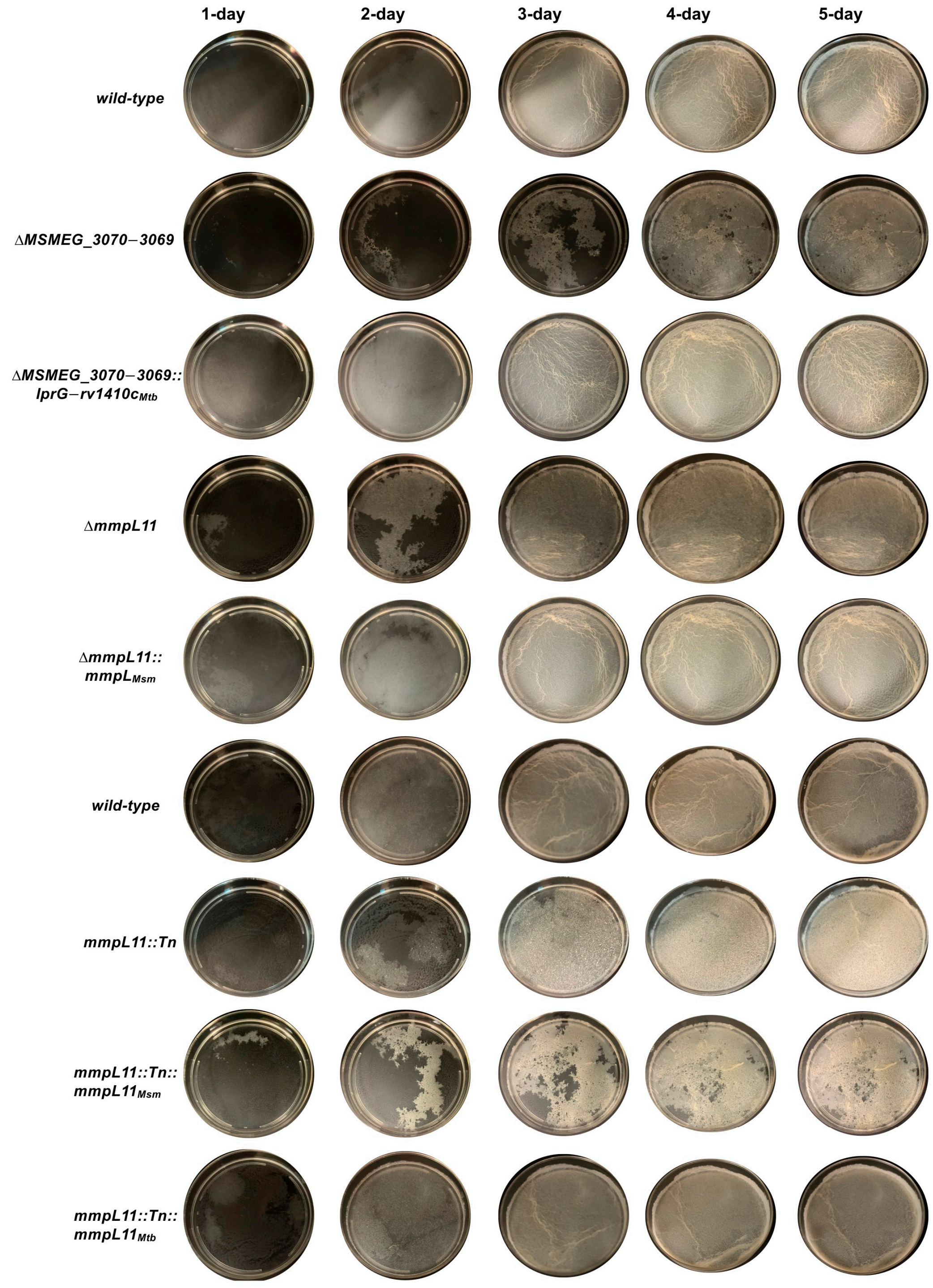

Figure S2. Five-day time course of pellicle biofilm formation at the air-liquid interface. Equal numbers of bacteria were inoculated into Sauton's medium without Tween 80 in polystyrene petri dishes and the plates were incubated at 30°C without disturbance for 5 days. Δ*MSMEG_3070−3069*, Δ*mmpL11*, *mmpL11::Tn* and *mmpL11::Tn::mmpL11*$_{Msm}$ strains were defective in pellicle biofilm formation starting at the day 2 time point. Data at days 2 and 4 are shown in Figure 1A. Data shown are representative of three biological replicates.

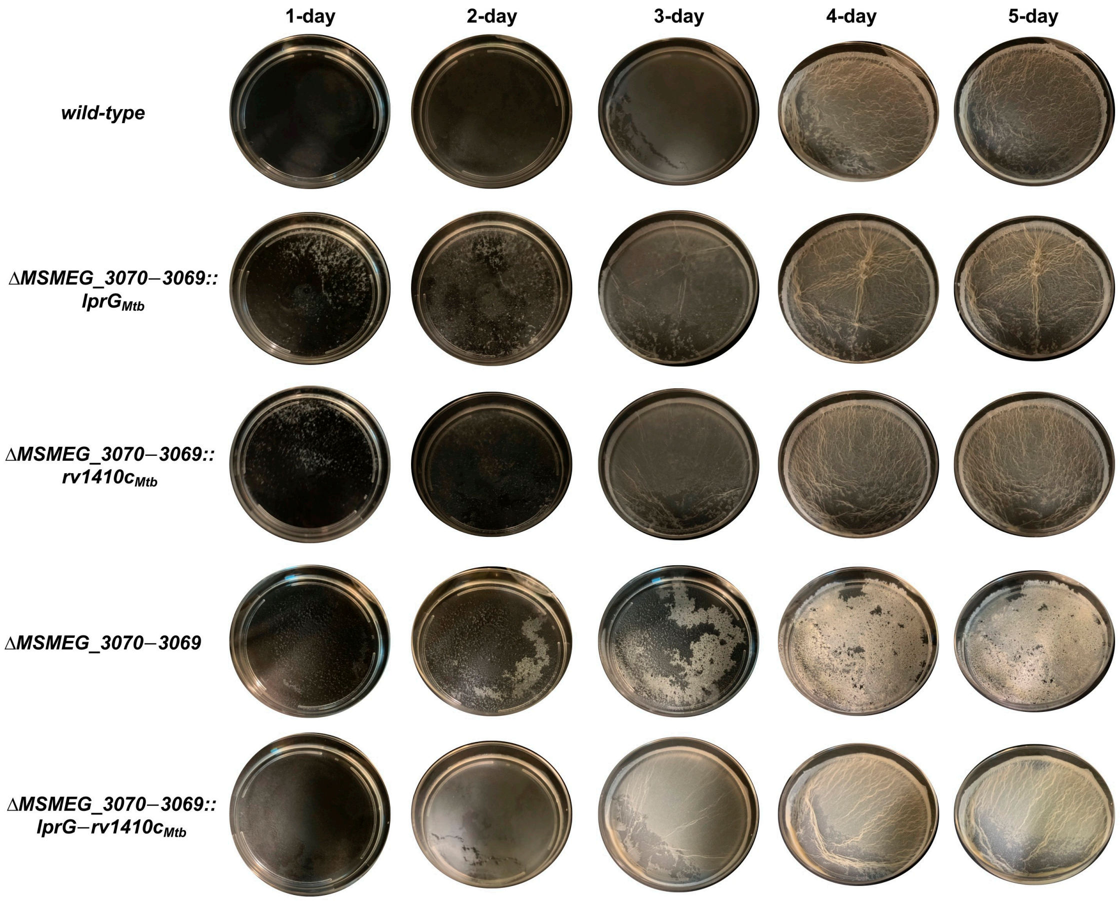

Figure S3. Five-day time course of *ΔMSMEG_3070−3069* mutant and complement strains pellicle biofilm formation at the air-liquid interface. Equal numbers of bacteria were inoculated into Sauton's medium without Tween 80 in polystyrene petri dishes and the plates were incubated at 30°C without disturbance for 5 days. Δ*MSMEG_3070−3069::lprG*$_{Mtb}$ and Δ*MSMEG_3070−3069::rv1410c*$_{Mtb}$ strains displayed wild type-like biofilm phenotype while *ΔMSMEG_3070−3069* strain was defective in pellicle biofilm formation starting at day 2. One biological replicate was performed as shown.

Table S1. Strains used in this study

Species	Genotype	Integration	Insert	Marker*	Source
Mycobacterium smegmatis mc[2]155	Wild type**				Farrow et al., 2008
	Δ*MSMEG_3070−3069*	none	none	none	Farrow et al., 2008
	Δ*MSMEG_3070−3069::rv1410c*	L5 attB	Phsp70−*rv1410c*	Zeo^R	Martinot et al., 2016
	Δ*MSMEG_3070−3069::lprG*	L5 attB	Phsp70−*lprG*	Zeo^R	Martinot et al., 2016
	Δ*MSMEG_3070−3069::lprG−rv1410c*	L5 attB	Phsp70−*lprG−rv1410c*	Zeo^R	Martinot et al., 2016
	Δ*mmpL11****	none	none	Hyg^R	This work
	Δ*mmpL11::mmpL11(MSMEG_0241)*	L5 attB	Pnative−*MSMEG_0241*	Hyg^R/Kan^R	This work
	Wild type**				Pacheco et al., 2013
	mmpL11::Tn	none	pVV16	Kan^R	Pacheco et al., 2013
	mmpL11::Tn:: $mmpLL1_{Msm}$	none	pVV16−*MSMEG_0241*	Kan^R	Pacheco et al., 2013
	mmpL11::Tn:: $mmpL11_{Mtb}$	L5 attB	Pnative−*mmpL11*	Hyg^R/Kan^R	Pacheco et al., 2013

*Zeo^R: Zeocin; Hyg^R: Hygromycin B; Kan^R: Kanamycin

** Lab-specific wild-type parent strains were shared by Dr. Eric Rubin for the *MSMEG_3070-3069* strains and Dr. Georgiana Purdy for the *mmpL11::Tn* strains.

*** Due to prevailing use in the literature, *mmpL11* is used to designate both the *M. tuberculosis* and *M. smegmatis* (*MSMEG_0241*) homologues.

Table S2. Plasmids and primers used in this study

Plasmid	Description
pJSC407	Mycobacteria knockout plasmid; Hyg^R
pMLP082	pJSC407 with 125 bp insertions from upstream and downstream of *mmpL11* (*MSMEG_0241*); Hyg^R
pNIT-RecEt-SacB-Kan	Plasmid with Che9c RecET gene for recombineering in mycobacteria; Kan^R
pMV306	Single-copy integrating plasmid with L5 integrase; integrates at mycobacteria chromosomal *attB* site; Kan^R
pMLP083	pMV306::*mmpL11* (*MSMEG_0241*) inserted via XbaI/ClaI sites; Kan^R
**Primer**	**Sequence**
**Gene deletion construct**	
omlp741	TGGATCCACGAAGCTTTGGTCAGAGCCTGGTTGGTC
omlp742	GGCCACCATGAAGCTTCTACAAGCGCATCATGAACTGCTGGATG
omlp743	CGGACAGGACTCTAGACTGGAGGAGGCGAAGTGACG
omlp744	CCGGGGATCCTCTAGAGCACGAGAACTTCCGACAG
**Complementation construct**	
omlp745	GATCTTTAAATCTAGAGTGTCCAGTTTCTTGCCTTGC
omlp746	ACTACGTCGACATCGATTCACTTCGCCTCCTCCAGCATTG
**PCR validation of gene deletion**	
ojcs240	CAGGCTCGCGTAGGAATCATC
**DNA sequencing**	
oevv137	GATGGCATAAAACGAAAGGCC
ojcs238	GCCTTTGAGTGAGCTGATACC
omlp336	CGCCTGATCAGGATCGTAATAC
omlp337	GACCTCGACGACCTGCAG
omlp338	CTGACGCTCAGTGGAACG
omlp051	CATCTTCGTGGACCTGGCC
omlp756	CTACCTCGCTCTCAACCAGTC
omlp757	GTCACCGGCATCTACCTCATC
omlp758	GATCAGCTCCCTGGACA
**RT-qPCR**	
olmn045	ACTGGAGACCGACGATCTGA
olmn046	GAGAACTTCCGACAGGGCTT
olmn021	CAGAAGGTCGGTATCCACGG
olmn022	ACGATCGGCATGTTCCACTT
olmn055	GTGACGTCGTGGTCATTCCT
olmn056	GGATCGGTAACGCAAATGGC
olmn023	TCGTGATGAAGCGTGAGGAC
olmn024	CAGACCGTTCTGGATCACCC
olmn047	TCGCGTCAAGCAGATCAAGT
olmn048	CTTCTTCAGGTCGGTCTCGG
olmn049	ACATCGAGAGCACGTGGAAG
olmn050	ATGTCCAGACGGGTCATGTG
olmn051	TGTCCAAGGGCATCCACATC

olmn052	GCGACGGTCATCCAGTTGTA
olmn025	ACCCGCAGCAGTTCATCTAC
olmn026	TGTTCGGATCGTTGGTCGAA
olmn027	AGTTCAGTTCCAGAGTGGCG
olmn028	CGGAGTCGAGGAACATCTCG
olmn029	CCCGGAGCAGTTCATCTACG
olmn030	GATGTTGACCATCGGGTCGT
ohvp088	GAAGACACCGACCTGGAACT
ohvp089	GACTCTTCCTCGTCCCACAC
